# Author Correction: Conventional CD4^+^ T cells present bacterial antigens to induce cytotoxic and memory CD8^+^ T cell responses

**DOI:** 10.1038/s41467-018-03074-6

**Published:** 2018-01-31

**Authors:** Aránzazu Cruz-Adalia, Guillermo Ramirez-Santiago, Jesús Osuna-Pérez, Mónica Torres-Torresano, Virgina Zorita, Ana Martínez-Riaño, Viola Boccasavia, Aldo Borroto, Gloria Martínez del Hoyo, José María González-Granado, Balbino Alarcón, Francisco Sánchez-Madrid, Esteban Veiga

**Affiliations:** 1 Department of Molecular & Cellular Biology, Centro Nacional de Biotecnología; Consejo Superior de Investigaciones Científicas (CNB-CSIC), Darwin 3, 28049 Madrid, Spain; 2 Hospital de Santa Cristina, Instituto de Investigación Sanitaria Princesa, 28009 Madrid, Spain; 3 Centro Nacional de Investigaciones Cardiovasculares Carlos III (CNIC), Melchor Fernández Almagro 3, 28029 Madrid, Spain; 4 Department of Cell Biology and Immunology, Centro de Biología Molecular Severo Ochoa (CBMSO); Nicolás Cabrera 1, Universidad Autónoma de Madrid, 28049 Madrid, Spain; 5 Instituto de Investigación Hospital 12 de Octubre (imas12), 28041 Madrid, Spain; 6 Hospital de la Princesa, Instituto de Investigación Sanitaria Princesa, 28006 Madrid, Spain

Correction to: *Nature Communications* 10.1038/s41467-017-01661-7, published online 17 November 2017

The original version of this Article contained an error in the spelling of the author José María González-Granado, which was incorrectly given as José María Gozález-Granado. This has now been corrected in both the PDF and HTML versions of the Article.

