# Supplementary material for: Conventional CD4+ T cells present bacterial antigens to induce cytotoxic and memory CD8+ T cell responses
Source: Nat Commun. 2017 Nov 17;8:1591. doi: 10.1038/s41467-017-01661-7 (PMC5691066; doi:10.1038/s41467-017-01661-7)
Supplement: Supplementary file 1 — Supplementary information [file 41467_2017_1661_MOESM1_ESM.pdf]

Supp. Figure 1

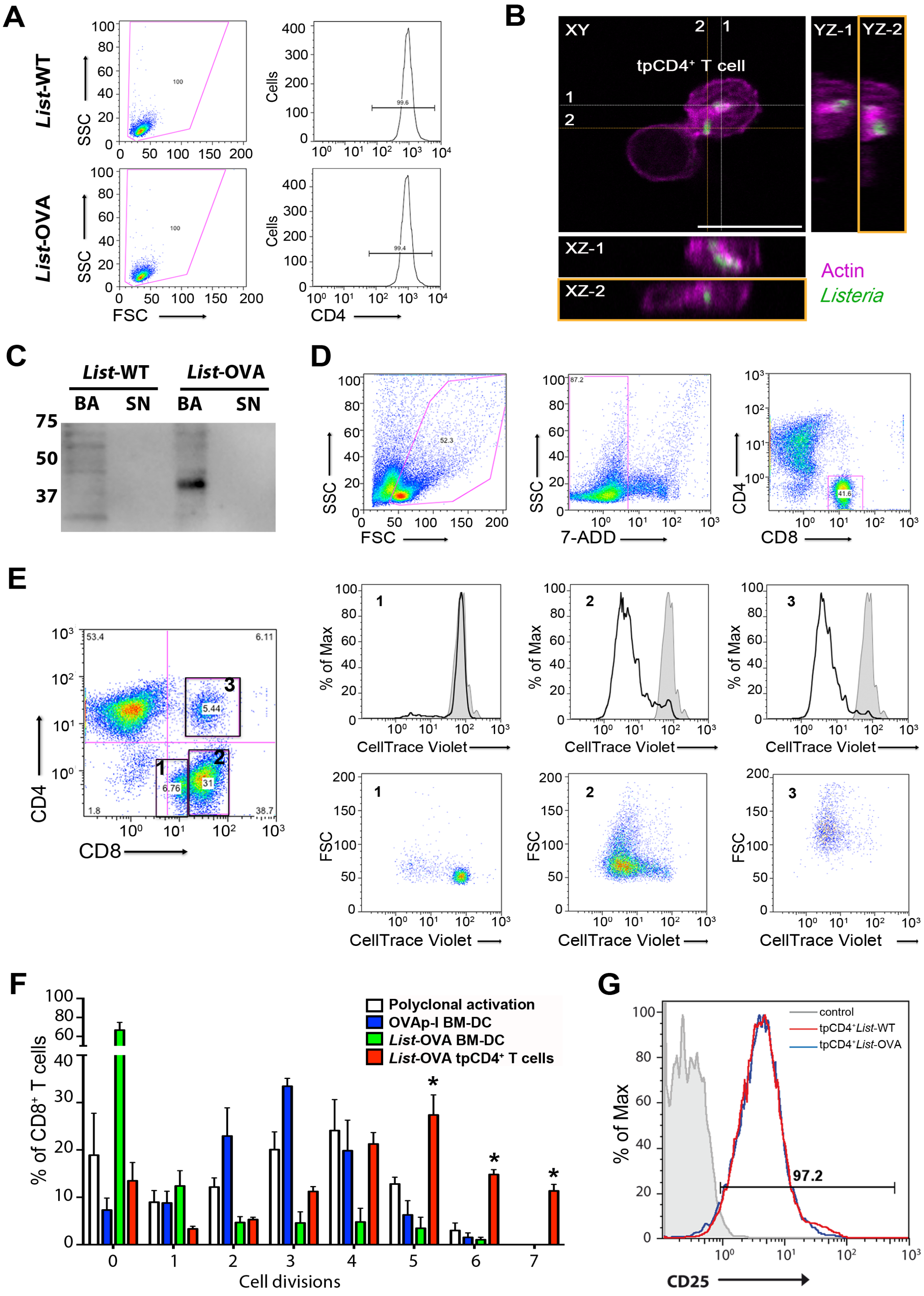

Supplementary Figure 1. **A,** Purity of *Listeria*-WT or *Listeria*-OVA tpCD4$^+$ T cells after cell sorting. Size and complexity are shown by plotting side- (SSC) vs forward-scattered (FSC) light (left panels). **B,** Orthogonal views of *Listeria* tpCD4$^+$ T cells purified at 3 h of conjugate formation. Actin is shown in magenta, and *Listeria* in green. XY together with YZ and XZ views (from lines marked in XY) are shown. Bar = 10 μm. **C**, OVA, bacteria-associated (BA) or in the concentrated supernatant (SN), detected by Western blotting. Bacteria tested were *Listeria*-WT (*List*-WT) or *Listeria*-OVA (*List*-OVA). Note that all original Western-blots together with the membranes stained for molecular size markers are provided at the end of the manuscript in Supplementary Fig. 5. **D**, Gating strategy for flow cytometry analysis to measure CD8$^+$ T cell proliferation induced by tpCD4$^+$ T cells. Only viable cells (7-AAD-negative) were analyzed. CellTrace Violet staining was analyzed in the CD8$^+$ population. **E,** *Listeria*-OVA tpCD4$^+$ T were incubated with naïve OT-I CD8$^+$ T cells (72 h). CellTrace Violet staining was analyzed in three CD8-positive populations: CD8^{+low} (1), CD8^{+high} (2) and CD4$^+$CD8$^+$ cell conjugates (3), which were also analyzed to detect blasts (activated T cells), which are larger than non-activated T cells (positive in FSC, forward scatter). **F**, Quantification of CD8$^+$ T cells proliferation after 3 days activation with anti-CD3/CD28 antibodies (polyclonal activation; white), OVAp-I-loaded BM-DC (blue), BM-DC infected with *Listeria*-OVA (green) or with *Listeria*-OVA tpCD4$^+$ T cells (red). The percentage of CD8$^+$ T cells that divided at indicated times is shown. * $p \leq 0.05$; two-way ANOVA with the Bonferroni post-test. Data shown as mean ± SD of four independent experiments. **G**, *Listeria*-WT (red line) or *Listeria*-OVA (blue line) tpCD4$^+$ T cell activation measured by CD25 expression. CD25 levels of non-activated lymphocytes is shown in grey.

Supp. Figure 2

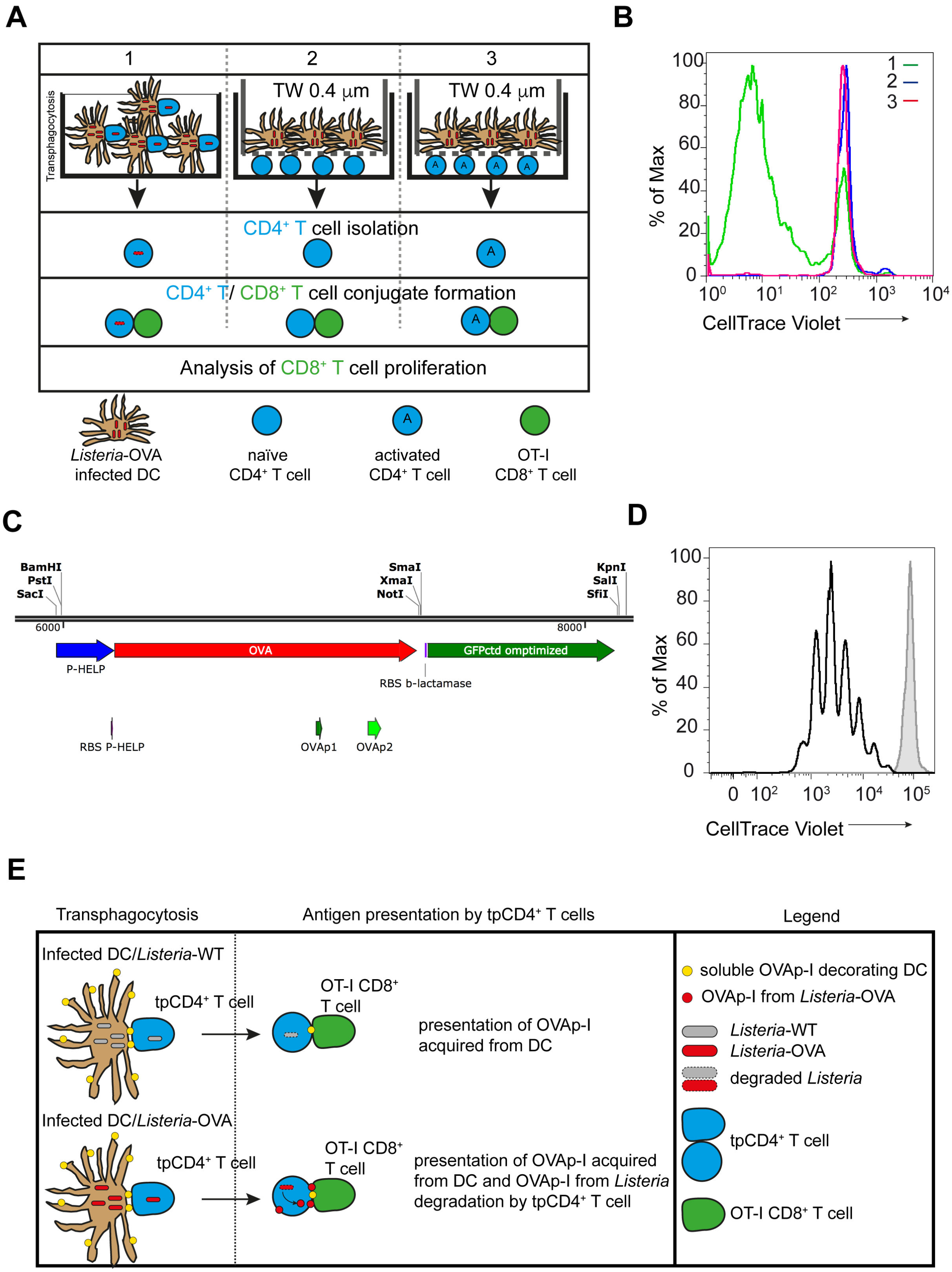

Supplementary Figure 2. A, Scheme, and **B** results of experiments to confirm the intracellular (bacteria) source of OVA. (A) *Listeria*-OVA-infected BM-DC were allowed to contact OT-II $CD4^+$ T cells (condition 1). Alternatively, *Listeria*-OVA-infected BM-DC were cultured on a polycarbonate barrier (0.4 μm pore size transwell; TW) that allowed passage of soluble material including proteins or exosomes, but impeded cell contact with non-activated (condition 2) or activated (anti-CD3/CD28; condition 3) OT-II $CD4^+$ T cells. After 16 h, $CD4^+$ T cells were re-isolated by cell sorting and used as APC to activate OT-I $CD8^+$ T cells. (B) OT-I $CD8^+$ T cell proliferation 4 days after contact with $CD4^+$ T cells as APC. Condition 1, green line; condition 2, blue line; condition 3, magenta line. **C**, OVA-GFP architecture of *Listeria*-OVA2. P-HELP promoter, RBS from each gene, OVAp (I and II) as well as the restriction enzymes allowing the removal of the genes are indicated. **D**, OT-I naïve $CD8^+$ T cell proliferation measured by CellTrace Violet staining 3 days after contact with *Listeria*-OVA2 (black line) tp$CD4^+$ T cells. Non-activated naïve $CD8^+$ T cells are shown in grey. **E**, Scheme of the experiment in Figure 2A. BM-DC were infected with *Listeria*-WT or *Listeria*-OVA and loaded with OVAp-II (to allow bacterial transphagocytosis to OT-II $CD4^+$ T cells) plus OVAp-I (the peptide that stimulates $CD8^+$ T cells from OT-I mice). OT-II $CD4^+$ T cells were incubated with these BM-DC to allow bacterial transphagocytosis; after 24 h, they were re-isolated by cell sorting and conjugated with naïve OT-I $CD8^+$ T cells. During conjugation, tp$CD4^+$ T cells might acquire MHC-I/OVAp-I from the DC membrane. If this was the main source of MHC-I/antigen that activates $CD8^+$ T cells, similar activation would be detected in both groups; if tp$CD4^+$ T cells process and present bacterial antigens via their own MHC-I, *Listeria*-OVA tp$CD4^+$ T cells would induce more potent $CD8^+$ T cell activation.

Supp. Figure 3

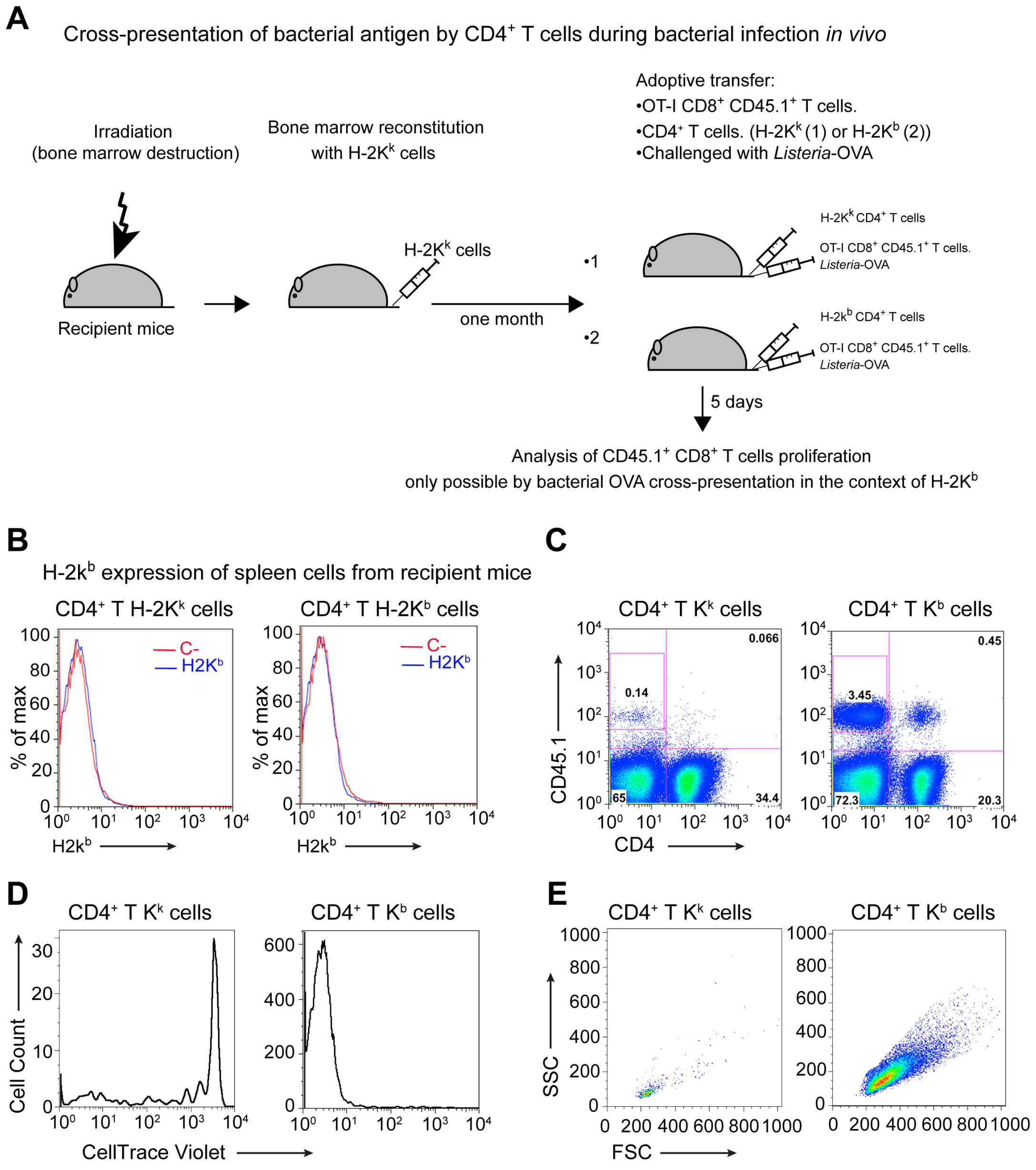

Supplementary Figure 3. A. Scheme of experimental approach used to determine whether CD4^{+} T cells present bacterial antigens in the course of an *in vivo* bacterial infection, to activate naïve OT-I CD8^{+} T cells. C57BL/6 mice were lethally irradiated and transplanted i.v. with bone marrow cells from H-2K^{k} mice. One month later, H-2K$^{k/k}$ CD4^{+} T cells (condition 1) or H-2K$^{k/b}$ CD4^{+} T cells (condition 2) were adoptively transferred, together with CD8^{+}CD45.1^{+} T cells from

OT-I mice and with MCC peptide. Recipient mice were also infected with *Listeria*-OVA (10^4 bacteria/mouse). At 5 days after infection, spleens were isolated from recipient mice and proliferation of $CD8^+$ T cells from OT-I mice (prestained with CellTrace Violet) was measured by flow cytometry. **B**. To confirm bone marrow elimination in recipient mice, we tested for H-$2K^b$ in spleen samples ($CD45.1^-CD4^-$). Red lines indicate secondary antibody background signal, blue lines indicate H-$2K^b$ expression. **C**, Dot plots showing the OT-I $CD8^+$ T cell ($CD45.1^+CD4^-$) population, analyzed 5 days after *Listeria*-OVA infection. **D**, Proliferation of the transferred OT-I $CD8^+$ T cells, 5 days after infection. **E**, Size and complexity of transferred OT-I $CD8^+$ T cells were analyzed in SSC-A and FSC-A to detect blasts (activated T cells). OT-I $CD8^+CD45.1^+$ cells were clearly activated in the mouse injected with H-$2K^b$ $CD4^+$ T cells (condition 2) and not in condition 1.

Supp. Figure 4

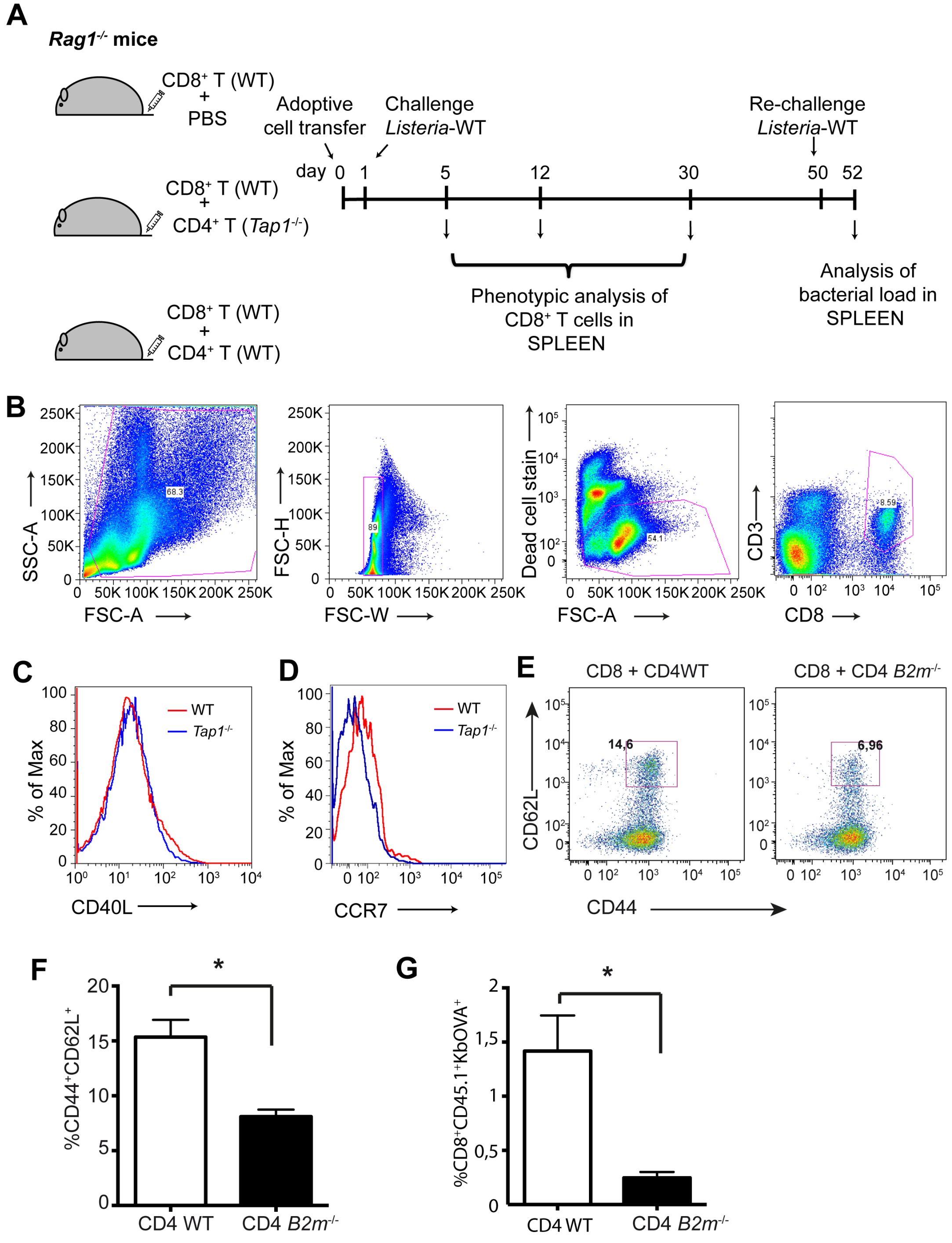

Supplementary Figure 4. **A**, Scheme of the experiment in Fig. 4. **B**, Gating strategy for flow cytometry analysis of memory CD8$^+$ T cells. All cells, excluding debris, were taken by size and

complexity (top left). From this selection, we gated for singlets (FSC-H vs FSC-W and SSC-H vs SSC-W) (top right, center left), then excluded dead cells (center right). Lymphocytes were analyzed by antibody staining for $CD3^+CD8^+$ (bottom). **C**, CD40L expression on polyclonal activated $CD4^+$ T cells from WT (red line) and *Tap1*$^{-/-}$ (blue) mice. **D**, CCR-7 levels on $CD3^+CD8^+CD44^+CD62L^{high}$ population from a mouse injected with $CD4^+$ WT cells (red line) or with $CD4^+$ *Tap1*$^{-/-}$ cells (blue line) at 30 days after *Listeria* challenge. **E-G**, Phenotypic analysis of memory $CD8^+$ T cell generation *in vivo*. *Rag1*$^{-/-}$ mice were adoptively transferred i.v. with WT $CD4^+$ T plus WT $CD8^+$ T cells (group 1) or with *B2m*$^{-/-}$ $CD4^+$ T plus WT $CD8^+$ T cells (group 2). The following day, all mice were challenged with *Listeria*-OVA2 (10^3 bacteria/mice; i.v.). 30 days after challenge, the $CD8^+$ T cell population from spleen was analyzed. (**E**), Representative dot plots showing CD62L and CD44 staining gated on the $CD45.1^+CD8^+$ T cell population. (**F**), Percentage of $CD62L^{high}$ $CD44^+$ gated on cells indicated in E (red outline). (**G**) Percentage of $CD45.1^+CD8^+$ T cells recognizing the H2-K^b/SIINFELK hexamer. Data shown as mean ± SD for 3 mice/group (group 1, white; group 2, black). * $p<0.05$ analyzed by unparied t test.

Supp. Figure 5

A

Original blots from figure 2 E,F

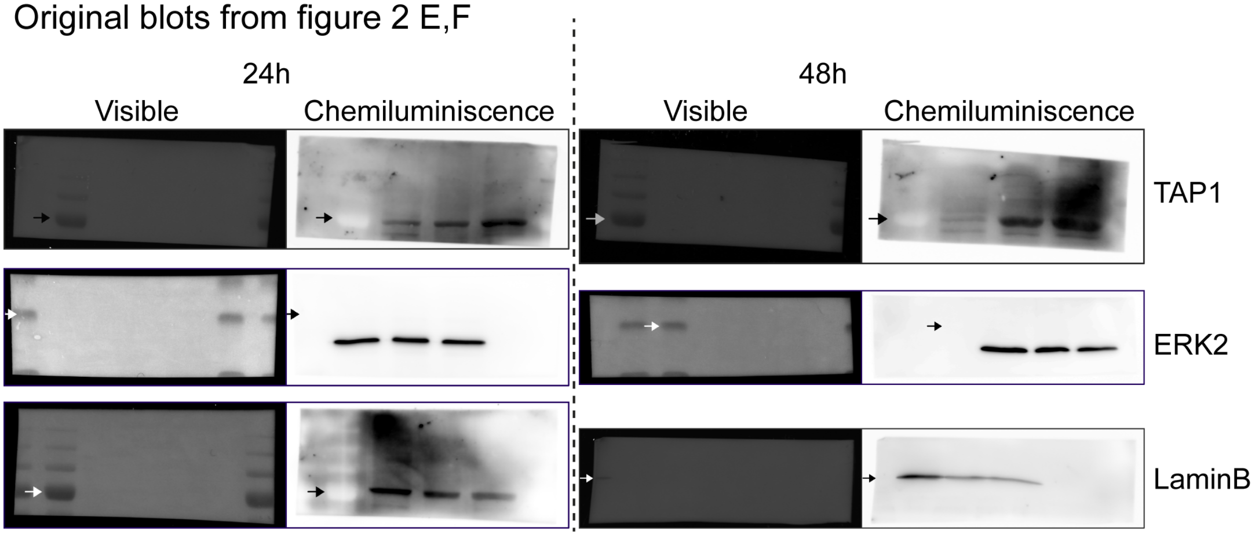

B

Original blots from Supp. figure 1C

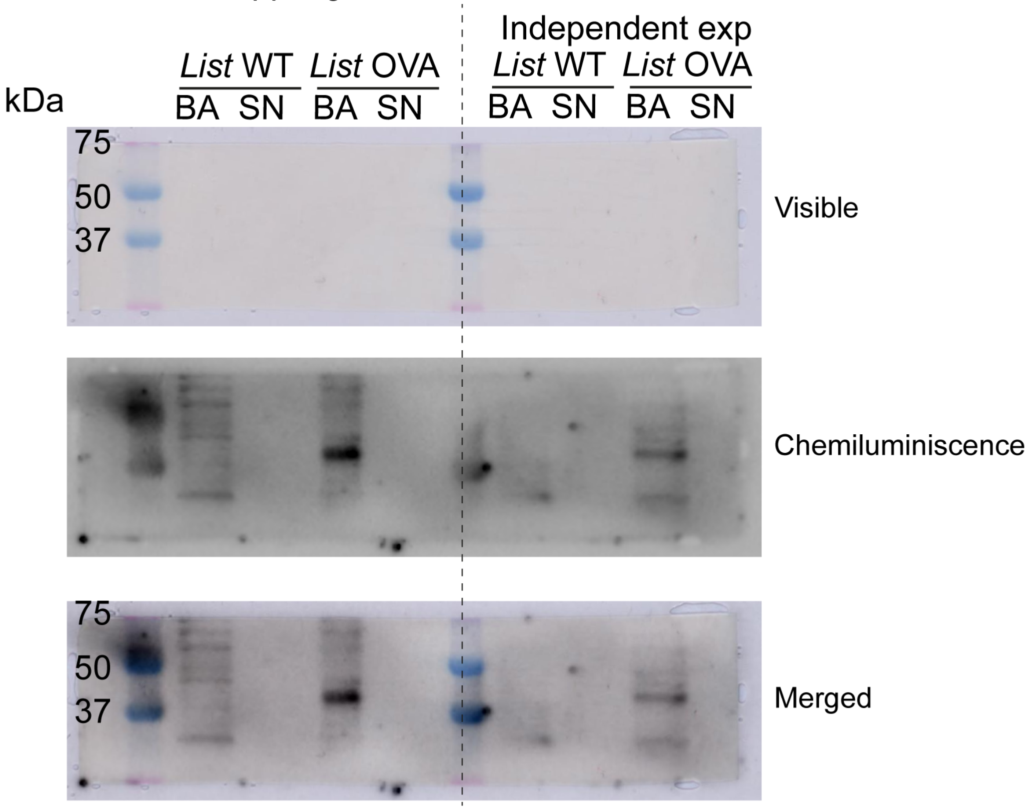

Supplementary Figure 5. Original blots. A, Original blots corresponding to Figure 2 E,F were visualized using visible light to detect the molecular size staining markers (Precision Plus Protein™ Dual Color Standards, #1610374; Bio Rad). The chemiluminiscence light emission from the HRP-conjugated secondary antibodies was detected on the same membranes. Arrows indicate the molecular size markers shown in Fig. 2E,F. **B,** Original blots corresponding to Supplementary Figure 1C. Molecular size staining markers (Precision Plus Protein™ Dual Color Standards, #1610374; Bio Rad) are shown in the "visible" panel. "Chemiluminiscence" panel shows the light emission of the HRP-conjugated secondary antibodies of the same membranes. We included here, in addition, the results from another independent experiment analysed in the same membrane. These last images were acquired using an *Amersham Imager 600* from *GE Health Care Life Sciences,* that allowed us to mount a merged image fusing the visible and the chemiluminiscence images.
