# Supplementary material for: Conventional CD4+ T cells present bacterial antigens to induce cytotoxic and memory CD8+ T cell responses
Source: Nat Commun. 2017 Nov 17;8:1591. doi: 10.1038/s41467-017-01661-7 (PMC5691066; doi:10.1038/s41467-017-01661-7)
Supplement: Supplementary file 3 — Description of Additional Supplementary Files [file 41467_2017_1661_MOESM3_ESM.pdf]

Description of Additional Supplementary Files

File Name: Supplementary Movie 1
Description: Three-dimensional rendering of confocal images showing an IS formed by a ListeriaOVA tpCD4+ T cell and a naïve CD8+ T cell from an OT-I mouse. The naïve CD8+ T cell is shown in grey and actin in cyan.
